# Recent Advances in Nose and Lung Organoid Models for Respiratory Viral Research

**DOI:** 10.3390/v17030349

**Published:** 2025-02-28

**Authors:** Lennart Svensson, Johan Nordgren, Åke Lundkvist, Marie Hagbom

**Affiliations:** 1 Division of Molecular Medicine and Virology, Department of Biomedical and Clinical Sciences, Linköping University, 581 83 Linköping, Sweden; johan.nordgren@liu.se (J.N.); marie.hagbom@liu.se (M.H.); 2 Division of Infectious Diseases, Department of Medicine, Solna, Karolinska Institute, 171 77 Stockholm, Sweden; 3 Zoonosis Science Centre, Department of Medical Biochemistry and Microbiology, Uppsala University, 751 05 Uppsala, Sweden; ake.lundkvist@imbim.uu.se

**Keywords:** human, nasal, lung, stem cells, organoids, respiratory virus

## Abstract

Studies on human respiratory viral infections and pathogenesis have historically been conducted using immortalized cells and animal models. However, these models are limited in their ability to recapitulate the complex structure of the human airway or the full spectrum of disease symptoms observed in humans. Recently, nose and lung organoids have revolutionized culture complexity in infection biology and have demonstrated potential for research on respiratory virus infections in humans. In this opinion, we review how advances in human nose and lung organoid models, which are able to express all cell types of the respiratory epithelia, i.e., Club, basal, goblet, and ciliated cells, have provided novel insight into the pathogenesis, age-dependent susceptibility, viral attenuation signature, and immune mechanisms of respiratory viruses such as SARS-CoV-2, respiratory syncytial virus, and influenza virus. The models have also demonstrated potential for studying hitherto uncultivable human viruses and to be useful for studies of zoonotic risk.

## 1. Introduction

Respiratory virus infections continue to pose a major threat to human health and account for most emerging infections. However, our understanding of its transmission and adaptation to humans remains limited [1]. A critical issue with predicting and assessing spillover risk and adaptation by respiratory viruses is the absence of biologically relevant models of assessment, despite the significant progress made in the fields of virology and epidemiology [2]. While the discovery of new viruses can help identify the animal origins of zoonoses, the value of these observations for postulating the risk of spillover or the potential for pandemic is limited [3]. Sequence analysis has provided important information about severe acute respiratory syndrome coronavirus 2 (SARS-CoV-2) evolution, but the mechanisms/factors relevant to human adaptation cannot be determined from the virus sequencing data alone [4]. While animal studies are critical for understanding the pathogenesis of emerging viruses, they often do not recapitulate the full spectrum of disease observed in humans. For example, none of the SARS-CoV-2 animal models involve acute respiratory distress symptoms [5]. Thus, non-transformed cell model systems that recapitulate the human tissue and cellular environment are necessary for understanding the transmission and adaption mechanisms of respiratory viruses [6].

Over the last few years, human stem cell-derived respiratory organoids have emerged as powerful tools for bridging the gap between transformed cell lines and in vivo human conditions. These airway organoid models are also included in the WHO’s list of tools for assessment of the pandemic risk of influenza [7,8].

**Figure 1:**
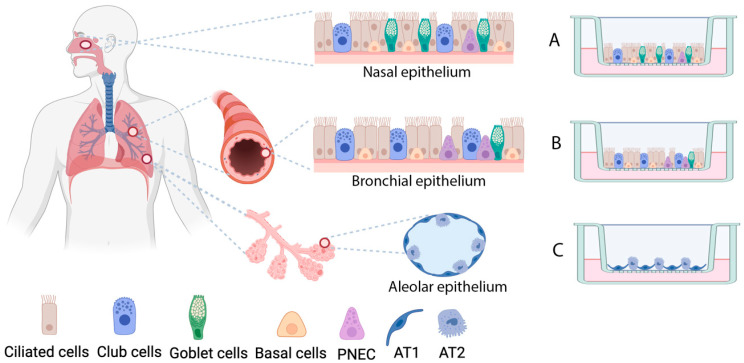
The main cell types in the respiratory tract and organoid air liquid interface organoid cultures. The upper respiratory epithelia in the nose have similar cell types as the bronchial epithelia and include the main cell types: ciliated cells, Club cells, goblet cells, basal cells, and rare pneumoendocrine (PNEC) cells. Despite the similarity of the epithelia, the composition, subtype, and morphology may differ along the respiratory tract. The cells of the alveoli, where the gas exchange occurs, contain alveolar type I (AT1) and type II (AT2) cells. Recent advances in organoid culture have made it possible to culture organoids from nose and different parts of the lung, and which can generate epithelia that mirrors in vivo. All three models can be generated from adult stem cells and bronchial and alveolar organoid models, as well as from induced pluripotent stem cells (iPSCs). Using air-liquid interface (ALI) culture, the apical parts of the epithelia are exposed to air, and mimic the in vivo situation well. ALI-cultures of nasal epithelia (**A**), bronchial epithelia (**B**), and alveolar epithelia (**C**) give unique opportunities for physiologically relevant studies of human respiratory airways, including at an individual level. Figure created in BioRender, modified from [14].

Several studies have demonstrated that these organoid models are highly physiologically relevant human disease models for respiratory viral infections [9,10]. Recent advances using these models have provided a breakthrough in our understanding of respiratory virus infections in humans [6,11] and can help to provide a better understanding of the pathogenesis of these viruses [9,12,13]. Here, we highlight recent important work that focusses on the application of human nose and lung organoid models in assessing infections caused by respiratory viruses and the power of the methodology to provide a better understanding of viral attenuation, pathogenesis, and age-dependent susceptibility. Organoids are self-organizing, three-dimensional (3D) in vitro culturing systems derived from stem cells, which replicate the in vivo architecture, functionality, and genetic signature of the original tissues [9]. Organoids can be generated from either somatic adult stem cells or pluripotent stem cells (PSCs), such as embryonic stem cells (ESCs) or induced pluripotent stem cells (iPSCs), which are somatic cells that have been reprogrammed into a pluripotent state [10]. ESCs are ethically controversial due to their derivation from embryos. While the reprogramming of somatic cells into iPSCs overcomes the ethical concerns associated with ESCs, iPSC-derived organoids require extensive stepwise differentiation protocols. In contrast, adult stem cells can be directly isolated from tissues or organ washes, allowing them to generate organoids that closely resemble their tissue of origin. Respiratory adult stem cells can generate cystic organoids within 3 to 10 days of culture, gradually increasing in size [11,12]. A non-invasive method for collecting nasal adult stem cells has been published [12], and more recently, bronchial and alveolar organoids have been cultured from adult stem cells in bronchoalveolar lavage (BAL) fluid, eliminating the need for tissue samples [11]. Due to their mature, tissue-specific phenotype, adult stem cell-derived organoids are useful for studying individual responses to viral infections. Organoids are cultured in 3D Matrigel and require a specialized culture medium to maintain their stem cell properties. They can be expanded, frozen, or used for differentiation into two-dimensional (2D) air–liquid interface (ALI) cultures or 3D tissue structures. In ALI cultures, the apical surface is exposed to air, mimicking in vivo conditions. Nasal and bronchial organoids develop into a complex, stratified epithelium containing all major cell types found in vivo (Figure 1). Secretory Club cells play a crucial role in immune defense by secreting the anti-inflammatory protein uteroglobin and contributing to tissue repair through their ability to generate new cell types [13]. Ciliated cells facilitate mucociliary clearance by trapping and expelling pathogens, while goblet cells secrete antimicrobial mucus that aids in mucociliary defense. Basal progenitor cells, which have self-renewing capabilities, can differentiate into various cell types. Alveolar organoids express alveolar type I (AT1) and type II (AT2) cells, similar to their in vivo counterparts [14]. AT2 cells secrete surfactants and have the ability to self-renew and differentiate into AT1 cells [14], which play a key role in gas exchange in the lungs.

## 2. SARS-CoV-2

As nasal epithelial cells are the first barrier in the respiratory tract and the entry point of respiratory pathogens, measurement of viral fitness in this organ can be a surrogate test for potential human infection and spread. In line with this notion, Wu et al. [15] used nasal epithelial organoids to investigate how SARS-CoV-2 entered the airway barrier of the mucus. They found that SARS-CoV-2 attaches to motile cilia via the angiotensin-converting enzyme 2 (ACE2) receptor and traverses the mucus layer, with the Omicron variant showing a heightened ability for cilia-dependent entry through the airway mucin barrier. Accordingly, depleting cilia was found to inhibit the entry of SARS-CoV-2.

Robinot et al. [16] investigated the functional and structural consequences of SARS-CoV-2 infection using a primary human bronchial epithelial model, which closely mimics the natural airway lining. They observed that SARS-CoV-2 primarily targeted ciliated cells, leading to significant damage and impaired motile function. These findings were further validated in an animal model. Using a similar model, Morrison et al. [17] found that SARS-CoV-2 attenuated IL-13, impacting viral entry, replication, and spread. Additionally, they noted aberrant ciliary organization and shedding, suggesting that the virus’s preference for ciliated cells and the resulting epithelial damage could impair airway clearance. These findings align with pathological observations from COVID-19 lung autopsies, which revealed extensive epithelial damage and cell shedding, exposing basal cells and contributing to airway obstruction [17]. A key observation was that SARS-CoV-2 exhibited a strong tropism for ciliated cells, while only 5% of infected cells were goblet cells [17]. While primary human epithelial organoid cultures typically lack immune cells, Choi et al. [18] developed adult human lung air-liquid interface organoids that retained epithelial and stromal architecture, along with lung-resident immune cells, including T, B, NK, and myeloid cells. Upon SARS-CoV-2 infection, these organoids exhibited an adaptive, virus-specific T cell response.

In 2022, Omicron rapidly replaced Delta as the dominant variant of SARS-CoV-2 [19]. The increased infectivity and transmission abilities of the Omicron variant may indicate a replication advantage in the upper respiratory tract. In fact, Ozono et al. [19] found that the Omicron variants enter and replicate more efficiently in the nasal epithelium than the D614G and Delta variants, possibly due to enhanced receptor interaction. Similarly, Chiu et al. [20] found, using nasal organoids, that the Omicron variant had higher infectivity and replicative fitness than prior variants, and that clinical SARS-CoV-2 samples from different patients exhibited variable replication capacity. In particular, the Omicron variant had higher infectivity than the Delta variant, with the wild-type strain (Wuhan) exhibiting the lowest infection rate. In another similar study, Tanneti et al. [11] investigated differences of SARS-CoV-2 variants in the human nasal model and found that the Delta variant was more cytopathic than the Omicron variant, with the latter favoring replication.

In support of the above observations, Li et al. [21] investigated the replicative fitness of BA.5 and earlier variants in human nasal organoids and found that the BA.5 subvariant exhibited dramatically increased replicative fitness and infectivity than the earlier wildtype and B.1.1.529 variant. With regard to host-dependent differences, Zhang et al. [22] found that the EG.5.1 and XBB.1.9.1 variants replicated more robustly in nasal organoids derived from a younger adult than in organoids derived from an older adult. All these findings are suggestive of functional differences among variants of concern at the cellular level, as well as distinct age-dependent mechanisms of pathogenesis in infected individuals. Importantly, these findings demonstrated that nasal organoids may hold promise in risk assessment of upcoming variants. A further elegant study by Beumer et al. [23], using CRISPR/Cas9-engineered organoids, identified essential host factors for coronaviruses whereas immortalized cells were found to have limited potential for coronavirus drug treatment investigations.

Breugem et al. [24] have shown that camelid nasal organoids are highly susceptible to Middle East respiratory syndrome coronavirus (MERS-CoV) infection, but not to infection with different SARS-CoV-2 variants (614G, BA.1, or EG.5.1.1). This study indicated that the camelid upper respiratory tract lacks expression of ACE2, which is associated with susceptibility to SARS-CoV-2 infection [24].

With regard to the use of nasal organoids for assessing zoonotic potential, Yang et al. [4] showed, using ex vivo lung tissues, human airway and nasal organoids, as well as primary human lung cells, that two animal coronaviruses, from the bat (BtCoV-WIV1) and the pangolin (PCoV-GX), shared similar cell tropism but exhibited a lower degree of replicative fitness in the human nasal cavity and airway tissue than SARS-CoV-2. Furthermore, the animal viruses triggered a milder proinflammatory response and resulted in a lower degree of apoptosis, while also exhibiting antagonist activity against interferon and the ability to partially escape adaptive immune barriers to SARS-CoV-2. The authors concluded that these animal viruses are not fully adapted to humans and, thus, carry a low spillover risk to humans [4]. In addition to infection and pathogenesis studies, there are other potential uses of nasal and lung models. For example, Montoya et al. [25] used a human nasal ALI epithelium as an in vitro system for assessing neutralizing antibody capacity to SARS-CoV-2.

Overall, the application of nasal and lung organoid models for SARS-CoV-2 studies have demonstrated great potential in elucidating: (i) characteristics of age-dependent differences in the pathogenicity of SARS-CoV-2 variants and their underlying mechanisms, (ii) the zoonotic potential of emergent variants and viruses, and (iii) the evaluation of neutralizing antibodies to SARS-CoV-2.

## 3. Respiratory Syncytial Virus (RSV)

RSV selectively targets ciliated cells in the human bronchial epithelium and uses Nucleolin as an entry coreceptor [26]. Studies using human bronchial epithelial cells by Griffiths et al. [27] revealed that the interaction between the pre-fusion RSV-F glycoprotein and the insulin-like growth factor-1 receptor (IGF1R) triggers the activation of protein kinase C zeta. These findings suggest a mechanism of viral entry in which receptor engagement and subsequent signal transduction facilitate the recruitment of a coreceptor to viral particles at the cell surface. RSV infection typically begins in the upper respiratory tract before extending into the lower respiratory tract. Although it does not result in lower respiratory infection in all cases, it is still fundamental to investigate differences between the various respiratory epithelia with regards to the infection capacity and pathogenesis of RSV. Rijsbergen et al. [28] compared the replicative fitness and innate responses of RSV A and B in nasal, bronchial, and small-airway tissue cultures and found that while both subgroups were able to replicate in the upper and the lower airways, subgroup A exhibited a replicative advantage particularly in nasal and bronchial cultures. Based on the findings, they concluded that airway organoids are a valuable model for studying RSV and can be used in the future to study factors that influence the disease severity of RSV.

A key question is how age influences RSV interactions with the bronchial epithelium and why young children experience more severe infections than older children. Zhao et al. [29] investigated this using lung and ALI cultures derived from infant and adult human donors. Their findings revealed that RSV spread extensively in the infant bronchial epithelium, leading to significant apoptotic cell death. In contrast, the adult bronchial epithelium exhibited limited RSV infection and remained structurally intact, showing no barrier damage. Similarly, Aloisio et al. [30] utilized the power of human nasal organoids in an elegant study to investigate why RSV infection in children is more severe than that in healthy adults. They found significant differences in how the infant and the adult epithelium responds to RSV infection. Infant-derived human nasal organoids were more susceptible to RSV replication and displayed stronger cytokine response, greater cellular damage, and enhanced mucous production than adult organoids. Based on their findings, they deduced that the epithelial cellular responses in infants may result in dysregulated innate epithelial immune responses that predispose them to more severe RSV infection than adults [30]. In another study, Rajan et al. [31] used a human nose organoid model to uncover and compare the pathogenic properties and therapeutic outcomes of RSV and SARS-CoV-2. SARS-CoV-2 induced severe damage to the cilia and the epithelium and minimal mucus secretion, with no interferon I response. In striking contrast, RSV induced hypersecretion of mucus and a profound interferon-λ response, along with ciliary damage [31].

To conclude, the studies so far imply that airway organoid models are beneficial for investigations of pathogenesis and therapeutic outcomes of RSV infections, as well as for revealing differences both between individuals and viruses. Future research would benefit from including immune cells in the model.

## 4. Influenza Virus

Influenza virus is primarily transmitted through the air, with the nasal epithelium serving as the entry point and primary site of infection. In the trachea, bronchi, and bronchioles, the virus predominantly attaches to the surface of ciliated epithelial cells, occasionally to goblet cells, and rarely to bronchiolar non-ciliated cuboidal cells [5]. A key factor in human influenza virus infection is the presence of epithelial cell receptors, specifically glycans terminated by an α2,6-linked sialic acid, which bind to human strains [6] and are found in the epithelial cells of the human nasal mucosa [7]. Studies have reported that influenza virus budding occurs at the tips of microvilli in the human airway epithelium [8].

Respiratory cultures derived from human nasal and lung cells are important models for studying tropism, replication kinetics, and host responses to influenza, and importantly, are included among the WHO tools for influenza pandemic risk assessment [7,8]. Zhou et al. [2] used 2D and 3D differentiated airway organoids, expressing serine proteases, which are essential for productive infection of human influenza viruses and low pathogenic avian influenza viruses. They showed that the known human infectivity of different influenza strains was recapitulated in the model. Hui et al. [7] found that the tropism and replication kinetics of human and avian influenza A viruses in human airway organoids mimicked those found in ex vivo cultures of human bronchus explants. Moreover, they found that airway lung organoids contained epithelial cell types that mimic the composition and cellular diversity found in vivo [7]. Another important observation was that the innate immune responses observed after infection of human airway organoids with several human and avian influenza virus strains mirror those seen in human ex vivo bronchus explants. Comparison of avian influenza strains showed that the highly pathogenic avian influenza H5N1 virus was only able to replicate in non-ciliated cells and exhibited a lower replication efficiency than the non-H5N1 viruses. Furthermore, organoids infected with the H5N1 virus had significantly higher expressions of interferon β, RANTES, and interleukin 6 than other subtypes of influenza. Thus, they were able to demonstrate the ability of this model to recapitulate the human airway system and to determine differences in the replication ability of H5N1.

Using bat airway organoids, Su et al. [32] investigated susceptibility to mammalian influenza strains and found that bat airway organoids derived from trachea or lung cells were highly susceptible to infection by two different porcine influenza A virus strains, namely H1N1 and H3N2. Interestingly, H1N1 exhibited lower virulence, but better replication and viral release than H3N2. Moreover, the bat airway cells expressed high amounts of α-2,3-linked sialic acid, the proposed receptor for avian influenza viruses, and only limited amounts of α-2,6-linked sialic acid, the proposed receptor determinant for mammalian influenza A virus strains. These observations indicated that the airway epithelium of bats does not have a barrier for interspecies transmission.

Thus, the studies on influenza viruses using airway organoid models have elucidated and compared the pathogenesis and immune responses to various strains, as well as demonstrated their utility in studies of interspecies transmission. The findings demonstrated that nasal and lung organoids are important tools to study zoonotic potential of different influenza strains as well as to assess their infection capacity, virulence, and immune response in humans.

## 5. Rhinovirus

While all rhinoviruses (RV) infect airway epithelial cells, RV-C utilizes a distinct host protein, cadherin-related family member 3 (CDHR3), to facilitate particle uptake [33]. The expression of CDHR3 is restricted to ciliated cells in both the upper and lower airway epithelium, which limits the cellular tropism of RV-C. Gagliardi et al. [33] have demonstrated that RV-C replication is confined to ciliated cells in human airway epithelium and is associated with the endoplasmic reticulum, inducing cytopathic effects.

The lack of propagation of certain human rhinoviruses in established cell lines has substantially hindered our understanding of its molecular mechanisms and pathogenesis. In contrast to human RV type A and B, type C rhinovirus is unable to infect and replicate in standard cell lines. After decades of non-successful efforts directed to overcome this limitation, Li et al. [13] recently demonstrated sustained propagation of human RV-C in nasal and lung organoids. Nasal organoids were found to be more susceptible to human RV-C than lung organoids, and lung organoids responded with a stronger innate immune response than nasal organoids. Through this work, the authors have demonstrated the power of respiratory organoids for studying previous uncultivable human viruses.

## 6. Conclusions and Future Perspective

Studies utilizing respiratory organoids have significantly advanced the modeling of respiratory diseases. Compared to animal models and immortalized cell cultures, often derived from non-respiratory tissues, human nasal and lung airway organoids more accurately mimic human tissue by incorporating multiple differentiated cell types and recapitulating the physiology of the human respiratory system [7]. One key advantage of human organoids is their ability to capture individual infection signatures, which may help in understanding host-specific responses. Several studies have demonstrated that these organoid models are biologically and physiologically relevant for studying human diseases [6,9,10]. For instance, human airway organoid cultures have produced influenza results comparable to those observed in human ex vivo bronchus cultures [7]. Similarly, Morrison et al. [17] found that SARS-CoV-2 infection disrupts ciliary organization and increases cilia shedding—findings consistent with pathological observations from autopsies of COVID-19 patients. Another example of host restriction is the requirement of CDHR3 on ciliated cells for rhinovirus type C (RV-C) infection in both the upper and lower airway epithelium, which limits the cellular tropism of RV-C [13,33]. While these examples highlight the relevance of respiratory organoids, future studies should incorporate immune cells and nerves to further enhance physiological complexity.

## Data Availability

Figure 1 was created with Biorender.com.
